# Effect of non-surgical periodontal therapy on the degree of gingival inflammation and stress markers related to pregnancy

**DOI:** 10.1590/1678-7757-2017-0630

**Published:** 2018-07-19

**Authors:** Fatma Ucan Yarkac, Ozge Gokturk, Osman Demir

**Affiliations:** 1 Gaziosmanpasa University, Faculty of Dentistry, Department of Periodontology, Tokat, Turkey.; 2 Abant Izzet Baysal University, Faculty of Dentistry, Department of Periodontology, Bolu, Turkey.; 3 Gaziosmanpasa University, Faculty of Medicine, Department of Biostatistics, Tokat, Turkey.

**Keywords:** Saliva, Stress, Gingivitis, Pregnancy

## Abstract

**Objectives:**

The purpose of this study was to determine the impact of nonsurgical periodontal therapy considering the salivary stress-related hormone and cytokine levels in the gingival crevicular fluid (GCF) on pregnant and nonpregnant women.

**Material and Methods:**

Thirty non-pregnant (control group) and 30 pregnant women (test group) that met the study inclusion criteria were chosen. Only participants with gingivitis were included. Clinical data and samples of GCF and saliva were collected at baseline and after periodontal therapy. The levels of interleukin-1 beta (Κ-1β) and IL-10, and concentration of salivary chromogranin A (CgA) hormone were analyzed by enzyme-linked immunosorbent assay (ELISA). The repeated measures analysis of variance was used for intragroup and intergroup analyses.

**Results:**

A major decrease in the gingival inflammation was observed in both groups after periodontal therapy (p<0.05). Periodontal treatment decreased the level of IL-1β in GCF (p<0.05) in control group, but no statistical difference was determined for GCF IL-1β in the test group. However, after periodontal therapy, the CgA hormone concentration was reduced in both groups (p<0.05). However, there was no difference in salivary CgA concentration, GCF IL-10 levels, and perceived stress scale (PSS)-10 between the groups (p>0.05).

**Conclusions:**

Within the limitations of this study, periodontal therapy significantly improved the periodontal status and stress level. In addition, the severity of the gingival inflammation during pregnancy was related to stress. However, further studies will be needed to substantiate these early findings.

## Introduction

Pregnancy gingivitis has been described as a prominent inflammatory reaction of the gingiva to the microbial dental plaque, which typically happens in the second and third trimesters of pregnancy^11^. The clinical and histological features of pregnancy gingivitis are similar to plaque-induced gingivitis^16^. Clinically, there is a moderate-to-severe inflammation, which can progress to severe hyperplasia, pain, and bleeding^16^. Different etiological pathways have been suggested in an attempt to understand the intense gingival inflammation. The most important theories to define pregnancy include hormonal effects on the immune system, the subgingival biofilm, the specific cells of the periodontium, and the vasculature. These potential etiologies have been studied in other articles^7^ ^,^ ^11^. However, there has been no consensus on the etiology of pregnancy gingivitis since periodontal complication is multifactorial in nature^19^.

According with the immune system theory, immunomodulatory alterations would render tissues in the periodontium more prone to gingival inflammation and release of proinflammatory cytokines during pregnancy^18^. Interleukin-1 beta (IL-1β), which has a major role in regulating the inflammatory reaction, is a multifunctional proinflammatory mediator^5^. Dinarello, et al.^5^ (1991) stated that the biological activity of IL-1β is multifunctional, activating acute-phase proteins, prostaglandins, and other cytokines as well as inducing collagen and collagenase production and bone resorption. Therefore, proinflammatory activity stimulates the action and coordinates the course of the immune response^14^.

Physiological or hormonal responses to pregnancy stress and the effect of pregnancy-specific stress are important issues. Physiological and hormonal changes in the body during pregnancy can affect the psychosocial state and increase susceptibility to stress^3^. Increased stress during pregnancy may lead to increased stress-related hormone levels^29^. Chromogranin A (CgA), one of the stress-related hormones, is released along with a glycoprotein under stress within sympathetic nerve endings and catecholamines from the adrenal medulla^2^. CgA is not only a marker of stress in the central nervous system, but it also participates in bacterial infections^2^. Metz-Boutigue, et al.^21^ (2003) reported that vasostatin-1, the N-terminal fragment of CgA, was released by animal polymorphonuclear (PMNs) lymphocytes during the stress process. Interestingly, PMN is the first step in the natural immune response to bacterial challenge in periodontal disease^10^. For this reason, CgA fragments are likely to be produced locally by human PMNs in periodontal disease^10^. Among these factors, interactions require a multi-dimensional stress concept including psychological, social, and physiological aspects and clarifying the stress research in pregnant women. To date, few researchers have investigated the effect of pregnancy on hormonal changes and the impact of pregnancy-specific stress on periodontal status^16^ ^,^ ^29^.

Psychosocial stress is known to affect periodontal status. Physiological and psychological changes in the body during pregnancy (weight gain, concerns of the mother about the baby, etc.) may affect the periodontal status. Therefore, the goal of this study is to examine the effect of stress state during pregnancy on periodontal status and the effect of non-surgical periodontal treatment on pregnant and non-pregnant women cytokines levels in gingival crevicular fluid (GCF) and on salivary stress-related hormone.

## Materials and methods

### Patient selection

This study was performed between June 2016 and January 2017 in the Periodontology Department of the Faculty of Dentistry, with approval from the Ethics Committee of the Gaziosmanpasa University. This study was registered at ClinicalTrials.gov (NCT03336957). Information on the purpose, content, and procedures was given verbally, in writing, and those that wanted to participate signed the informed consent form. The study protocol was performed in accordance with the relevant guidelines of the Declaration of Helsinki.

**Figure 1:**
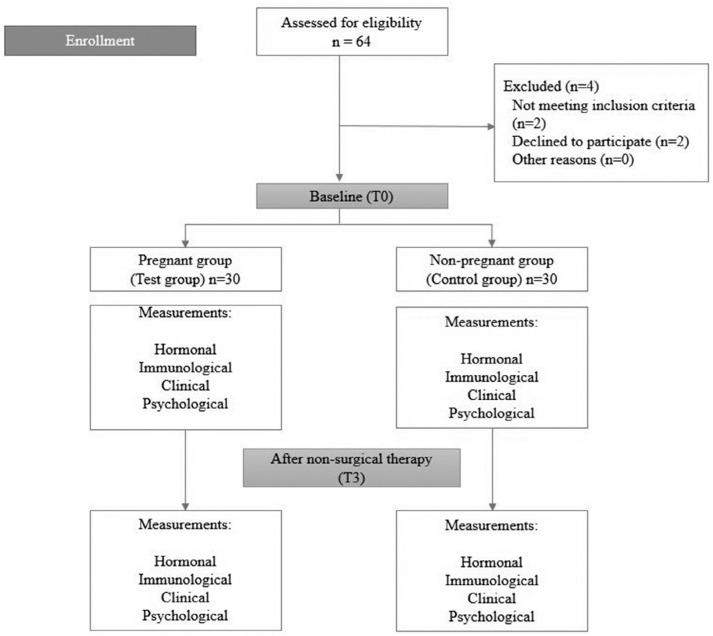
Follow-up flowchart of pregnant and non-pregnant women

According to the power analysis, the association between stress and gingival inflammation in pregnancy with an 80% power was determined at a minimum of 60 patients. A total of 64 patients were included. From the 64 female patients (age range 20 to 45), two refused to participate and two did not meet the inclusion criteria. As a result, 60 patients were included in the study (Figure 1).

The patients were split into two groups according to their pregnancy status: pregnant (test) and nonpregnant (control) groups. Pregnant women in the test group were in the 23±2 gestational weeks. The menstrual cycle was controlled by scheduling the visits of non-pregnant women during the luteal phase (days 17-21) of the cycle.

Inclusion criteria consisted of: 1) clinical diagnosis of gingivitis; 2) probing pocket depth (PPD) ≤3 mm in all four quadrants; 3) presence of at least 20 teeth; and 4) generally systemically healthy. Gingivitis was diagnosed based on clinical and radiographic criteria (only clinical criteria for pregnant patients). The diagnosis was made if the inflammation was confined to the gingiva and teeth with no attachment loss. Exclusion criteria consisted of: 1) use of antiinflammatory, antimicrobial and hormone therapy within the preceding 6 months; 2) presence of psychiatric disorders, systemic disease and smoking cigarettes; and 3) breastfeeding, pregnancy and menstrual periods (for the control group).

### Psychological measurements

Patients completed the Turkish version of the perceived stress scale (PSS)-10 before periodontal therapy^6^. The patient was given a period of time in a calm environment to complete the scale form. The internal consistency coefficient of the Turkish PSS-10 was 0.82 and the test-retest reliability coefficient was 0.88^6^. PSS-10, a self-report scale comprising 10 items, consisted of descriptive terms of stress symptoms rated by the subject on a four-point scale. The total score classified the stress density levels: less than 9, low stress; 9 to 16, moderate stress; and ≥16, high stress^26^. In addition, a questionnaire was used to ask the patients in test and control groups about their attitude to life and the stress induced by their families^20^.

### Clinical examination and periodontal therapy

Periodontal clinical measurements and periodontal treatment were applied by same examiner. Ten randomly selected patients that were not included in the study were evaluated to estimate the reliability of the periodontal measurements before implementation. The intra-class correlation coefficient (ICC) was 0.92±0.04 and 0.97±0.03 for clinical attachment level (CAL) and probing pocket depths (PPD) measurements, respectively. Clinical parameter measurements, including PPD (the distance from the gingival margin to the base of the gingival sulcus), CAL, full mouth plaque index (PI)^28^, and gingival index (GI)^17^ scores were registered using a periodontal probe after saliva and GCF sample collection. All clinical measurements were examined from six sites *per* tooth, excluding third molars. Then, non-surgical periodontal therapy (NPT), which consisted of scaling and oral hygiene instruction, was applied in a single session. Clinical measurements, collection of GCF and saliva samples were repeated after three weeks.

### GCF and saliva sample collection

The anterior teeth selected for GCF sampling were isolated from the saliva using cotton rolls, and a gentle stream of air was applied parallel to the root surface for 5 to 10 seconds to dry the area. The paper strips (Periopaper, Oraflow Inc., Amityville, NY, USA) were gently inserted into the gingival crevice until resistance was felt, left in place for 30 seconds, and collected from mesiobuccal sulcus of teeth in the anterior region of each patient (two samples *per* patient and *per* visit). The samples containing blood were discarded and were placed in sterile tubes. Then, GCF samples were immediately frozen at −20°C until the assay procedure.

Samples of saliva were collected from the spit of all volunteers before applying the periodontal clinical parameter measurements. Morning hours were preferred for sample collection not to affect hormone results and subjects were requested not to eat, brush, drink, or chew gum within 90 min to avoid contamination. The samples were always taken at the same time and put into polypropylene tubes. Then, all samples were centrifuged at 3,220 rpm for 10 min and immediately frozen at −20°C until the assay procedure^27^.

### Laboratory assessment

All samples were evaluated using enzyme-linked immunosorbent assay (ELISA) according to the manufacturer's instructions. Cytokine levels in the GCF were evaluated using IL-1β and IL-10 ELISA kits (EBioscience, Biomedical Research Center, San Diego, CA, USA), and the salivary CgA hormone concentration was evaluated using CgA ELISA kit (YK070 Human CgA EIA kit, Biovendor Laboratory, Asheville, NC, USA). Results were calculated using the standard curves formed by each assay for saliva and GCF volume and were given in pmol/mL and pg/mL, respectively.

### Statistical analysis

The clinical data were analyzed using statistical software (IBM SPSS Statistics 19, SPSS Inc., Somers, NY, USA) and were expressed by mean and standard deviation. The difference between the two means for the continuous variables was tested for significance using one-way analysis of variance (ANOVA). Two-way repeated measures ANOVA was used for repeated measures. The relationship between qualitative variables was examined by Chi-square test. Pearson's correlation coefficient was used for the relationship between qualitative variables. A p-value less than 0.05 was considered statistically significant.

## Results

Demographic and socioeconomic variables of the study as means and standard deviations are indicated in Table 1. There were no differences between groups in terms of all demographic and socioeconomic variables at baseline (p>0.05). In Table 2, the clinical data, including means of the gingival index, plaque index, clinical attachment levels and probing on depth scores, and at baseline and after NPT are shown. Periodontal clinical parameters decreased statistically after therapy in both groups (p<0.05). In the test group, periodontal probing depth index, gingival index, and plaque index scores were higher than the control group (p<0.05).

**Table 1:** Demographic and socioeconomic variables in non-pregnant and pregnant women

Variable	Preg. group (n=30)	N-Preg. group (n=30)	p
Age (mean±SD)	28.93±4.04	27.93±6.61	0.483
Number of teeth	25.66±1.97	26.13±1.22	0.387
Education level, n (%)			
Primary school	8 (26.7)	5 (16.7)	
Secondary school	2 (6.7)	3 (10.0)	0.485
High school	8 (26.7)	5 (16.7)	
University	12 (40.0)	17 (56.7)	
Socioeconomic level (monthly income level, Turkish Liras, n (%)			
Low (<1.400)	5 (16.7)	8 (26.7)	
Medium (2.500-5.000)	9 (30.0)	7 (23.3)	0.614
High (>5000)	16 (53.3)	15 (50.0)	
Frequency of tooth brushing, n (%)			
Frequent (two or tree times *per* day)	8 (26.7)	9 (30.0)	0.874
Infrequent (others)	22 (73.3)	21 (70.0)	
Family-induced stress, n (%)			
Yes	5 (16.7)	3 (10)	0.448
No	25 (83.3)	27 (90)	

* p<0.05

**Table 2:** Periodontal characteristics of patients within the groups at baseline (T0), at the end of treatment (T3) and between TO and T3

Variable		T0			T3		T0/T3-Pr	T0/T3-N-Pr
	Preg. group Mean±SD	N-Preg. group Mean±SD	pa	Preg. group Mean±SD	N-Preg. group Mean±SD	pb	pc	pd
PPD	2.43±0.44	2.25±0.37	0.093	1.82±0.39	1.60±0.26	0.014*	0.000**	0.000**
CAL	2.23±0.19	2.05±0.42	0.192	1.93±0.24	1.64±0.14	0.026*	0.000**	0.000**
PI	1.72±0.75	2.02±0.75	0.139	1.25±0.87	0.41±0.61	0.000**	0.016*	0.000**
GI	1.80±0.69	1.58±0.72	0.230	0.95±0.55	0.34±0.44	0.000**	0.000**	0.000**

SD: standard deviation; pa: statistical difference between Preg. and N-Preg. groups at T0; pb: statistical difference between Preg. and N-Preg. groups at T3; pc: statistical difference between T0 and T3 for the Preg. group; pd: statistical difference between T0 and T3 for the N-Preg. group.

** p<0.001

* p<0.05

Table 3 shows the cytokine levels in the GCF throughout the study. There were no differences in levels of GCF IL-10 and IL-1β levels at baseline (p>0.05). Regarding IL-10 levels, there were no differences between groups during the study (p>0.05). In addition, GCF IL-1β cytokine levels had a statistically significant reduction in the control group. However, there was no difference for GCF IL-1β level in the test group after three weeks. There were no statistically significant differences for IL-1β levels in the test group compared to the control group at baseline, but there was a higher statistically significant difference after three weeks (p<0.05). CgA levels in saliva and PSS- 10 results throughout the study are shown in Table 3. There were no differences in the concentration of CgA and in PSS-10 scores between groups throughout the study (p>0.05). Furthermore, the concentration of the CgA hormone significantly reduced among groups after therapy (p<0.05).

**Table 3:** Gingival crevicular fluid (GCF) cytokines levels and stress markers in patients within the groups at baseline (T0), at the end of treatment (T3), and between T0 and T3

Variable	T0			T3			T0/T3-Pr	T0/T3-N-Pr
	Preg. group Mean±SD	N-Preg. group Mean±SD	pa	Preg. group Mean±SD	N-Preg. group Mean±SD	pb	pc	pd
IL-1β (pg/mL)	44.94±34.21	64.79±46.11	0.063	43.79±32.83	25.62±13.08	0.007**	0.893	0.001**
IL-10 (pg/mL)	9.62±6.84	7.65±7.30	0.285	9.68±9.04	10.29±7.04	0.770	0.973	0.155
CgA (pmol/mL)	2.33±0.32	2.20±0.31	0.120	2.11±0.34	1.98±0.48	0.235	0.011*	0.011*
PSS-10	18.37±5.24	15.9±5.72	0.087	16.10±5.80	17.27±4.29	0.379	0.757	0.092

SD: standard deviation, pa: statistical difference between Preg. and N-Preg. groups at T0; pb: statistical difference between Preg. and N-Preg. groups at T3; pc: statistical difference between T0 and T3 for the Preg. group; pd: statistical difference between T0 and T3 for the N-Preg. group.

* p<0.05

** p<0.001

## Discussion

This analysis was performed to define the impact of non-surgical periodontal therapy in pregnant and nonpregnant women considering cytokines levels in the gingival crevicular fluid and on salivary stress-related hormone. NPT successfully reduced the clinical signs of gingival inflammation for salivary CgA concentration, and GCF IL-1β levels in the test and control group. However, it did not increase GCF IL-10 levels. There were no statistically significant differences at IL-1β levels in the pregnant group compared to the nonpregnant group at baseline; however, the IL-1β level in the pregnant group was significantly higher after three weeks. Furthermore, this study shows that pregnant women have deeper periodontal pockets, more gingival inflammation, and higher CgA level than non-pregnant women after periodontal treatment. The high level of gingival inflammation may be explained by changes in the immune system and by hormonal and psychological changes during pregnancy.

Pregnancy increases the susceptibility to gingival inflammation. The increase in clinical manifestations of gingival inflammation in pregnancy is consistent with the molecular changes in inflammatory mediator levels in GCF. In some studies, there was no association between the IL-1β levels in GCF and the increased gingival inflammation during pregnancy^4^ ^,^ ^7^. Figuero, et al.^7^ (2010) found that no changes in IL-Ιβ and PGE_2_ levels were observed in pregnant women without periodontitis. However, there are also studies that do not support this idea. Fiorini, et al.^8^ (2013) investigated 60 pregnant female patients with periodontitis to check whether their serum and GCF cytokine levels changed with periodontal treatment. They found that IL-8 and IL-1β levels in GCF were significantly decreased in serum cytokine levels after periodontal therapy, although there was no change for IL-6, IL-10, IL-12, p70, and TNF-α cytokine levels in GCF. Kaur, et al.^15^ (2014) found that the levels of GCF, IL-1β, and IL-6 in pregnant women with periodontitis were reduced after periodontal therapy. Michalowicz, et al.^22^ (2009) submitted that IL-8, IL-6, IL-1β, and TNF-a levels, which are serum inflammatory markers, did not change with the NPT in pregnant women diagnosed with periodontitis. In the present study, there was a major reduction in doses of IL-1β after treatment for non-pregnant women, but there was no statistically significant decrease in the test group. Decreased levels of IL-1β after treatment in non-pregnant individuals were parallel to the periodontal healing observed in these women. The reason the test group had a higher level of IL-1β compared to the control group after treatment may be due to less periodontal recovery in pregnant women^1^. These differences among the groups of cytokine levels seem to result from hormonal and physical changes observed during pregnancy. Pregnancy may decrease host response and increase the risk of severe periodontal disease. It may also increase individual susceptibility to infection by reducing the immune system function its response by inhibiting lymphocyte, macrophage, and monocyte functions, which cause chronic inflammation^12^.

Physiological and hormonal changes experienced by women during pregnancy may also increase the sensitivity of stress^29^. Stress in pregnancy leads to increased levels of stress-related hormones^29^. CgA, a stressed-related hormone, is a clinical marker of neuroendocrine cells and is an important indicator of sympathoadrenal activity^23^. Some studies have indicated that salivary CgA levels are elevated in several stressful conditions, such as mouth opening and exposure to traffic noise. However, a reduction in stress-relieving events such as lavender inhalation or exposure to negative air ionization can combat this affect^10^ ^,^ ^21^. Furthermore, it has been declared that CgA is also associated with periodontal disease^13^. Haririan, et al.^13^ (2012) demonstrated that salivary cortisol, CgA, and alpha amylase (AA) levels were altered in individuals with different periodontal diseases. CgA and AA have been implicated in altering proinflammatory and anti-inflammatory cytokine production and have been shown to affect periodontal disease activity. Parekh and Putney^24^ (2005) reported stress-stimulated PMN secretion by the release of CgA and its derivatives from adrenal medullary. The relationship between periodontal disease and salivary stress markers such as CgA, AA, cortisol, and β-endorphin investigated in previous studies have been reported, concluding that stress influenced periodontal status with physiological and behavioral mechanisms^13^ ^,^ ^25^. Contrary to other studies, Forte, et al.^9^ (2010) suggested that there is no effect of the psychosocial stress on salivary peptides in periodontal disease. In the present study, there was no statistically significant difference in salivary CgA concentration at baseline and after treatment between the groups. Furthermore, salivary CgA concentration was significantly reduced in both groups. These findings suggest that stress levels decreased due to periodontal healing. Furthermore, there was a significant decrease in the control group after periodontal therapy, and no significant decrease in IL-1β levels of the test group. These findings may explain, at least in part, that the reduction of GCF IL-1β levels in pregnant women may not be associated with stress, and the reduction of GCF IL-1β in non-pregnant women may be associated with stress.

This study has some limitations. Stress is a versatile concept and additional markers that are known to be associated with stress need to be examined in assessing stress. Also, the psychological state of pregnancy is highly variable and the evaluation of acute and chronic stresses will provide more accurate results than assessing the effect of stress. Additionally, the assessment of gingival disease requires postpartum periodontal status, this study has a limited sample size, and the findings need to be proved in the future by clinical trials.

## Conclusions

Within the limitations of this study, periodontal therapy significantly reduced the stress-related hormone level and ensured a significant decrease in clinical periodontal parameters. Also, the severity of the gingival inflammation during pregnancy was believed to be related to stress. This finding should be interpreted with caution since more prospective studies are needed to prove the effect of non-surgical periodontal therapy on gingival inflammation status and on salivary stress-related hormones during pregnancy.
